# Analysis of Hepatitis C Virus Decline during Treatment with the Protease Inhibitor Danoprevir Using a Multiscale Model

**DOI:** 10.1371/journal.pcbi.1002959

**Published:** 2013-03-14

**Authors:** Libin Rong, Jeremie Guedj, Harel Dahari, Daniel J. Coffield, Micha Levi, Patrick Smith, Alan S. Perelson

**Affiliations:** 1 Department of Mathematics and Statistics and Center for Biomedical Research, Oakland University, Rochester, Michigan, United States of America; 2 Theoretical Biology and Biophysics, Los Alamos National Laboratory, Los Alamos, New Mexico, United States of America; 3 University Paris Diderot, Sorbonne Paris Cite, 75018 Paris, France; 4 INSERM, UMR 738, 75018 Paris, France; 5 Department of Medicine, University of Illinois at Chicago, Chicago, Illinois, United States of America; 6 Department of Medicine, Loyola University Chicago, Chicago, Illinois, United States of America; 7 University of Michigan-Flint, Mathematics Department, Flint, Michigan, United States of America; 8 Clinical Pharmacology, Pharma Research and Early Development, Roche, Nutley, New Jersey, United States of America; Imperial College London, United Kingdom

## Abstract

The current paradigm for studying hepatitis C virus (HCV) dynamics in patients utilizes a standard viral dynamic model that keeps track of uninfected (target) cells, infected cells, and virus. The model does not account for the dynamics of intracellular viral replication, which is the major target of direct-acting antiviral agents (DAAs). Here we describe and study a recently developed multiscale age-structured model that explicitly considers the potential effects of DAAs on intracellular viral RNA production, degradation, and secretion as virus into the circulation. We show that when therapy significantly blocks both intracellular viral RNA production and virus secretion, the serum viral load decline has three phases, with slopes reflecting the rate of serum viral clearance, the rate of loss of intracellular viral RNA, and the rate of loss of intracellular replication templates and infected cells, respectively. We also derive analytical approximations of the multiscale model and use one of them to analyze data from patients treated for 14 days with the HCV protease inhibitor danoprevir. Analysis suggests that danoprevir significantly blocks intracellular viral production (with mean effectiveness 99.2%), enhances intracellular viral RNA degradation about 5-fold, and moderately inhibits viral secretion (with mean effectiveness 56%). The multiscale model can be used to study viral dynamics in patients treated with other DAAs and explore their mechanisms of action in treatment of hepatitis C.

## Author Summary

Chronic infection with hepatitis C virus (HCV) remains an important health-care problem worldwide despite significant progress in the development of HCV therapy since the discovery of the virus in 1989. Current treatment options are focused on direct-acting antiviral agents (DAAs) that target specific steps of the HCV life cycle. Danoprevir, one of the DAAs that inhibit the HCV NS3-4A protease, has induced substantial viral load reductions in patients receiving therapy. We study the viral decline during therapy using a multiscale age-structured model that accounts for the dynamics of intracellular viral replication, and which includes the major steps in the HCV life cycle that are targeted by DAAs. We examine the biological parameters contributing to different phases of the viral decline after treatment initiation. We also explore the mechanisms of action of danoprevir and estimate its treatment effectiveness. The multiscale model provides a theoretical framework for studying virus dynamics in hepatitis C patients treated with other DAAs currently in clinical development, and may help one to optimally combine drugs with complementary modes of action to maximize the HCV cure rate.

## Introduction

Hepatitis C virus (HCV) infection is a major cause of chronic liver disease and a leading reason of liver transplant in the world. About 130–170 million people are chronically infected with HCV [1]. Achieving a long-term sustained virologic response (SVR), defined as an undetectable HCV RNA level in serum 24 weeks after the end of treatment, is the most effective way to prevent disease progression [2]. Until 2011, the standard of care for HCV infection has been the combination of weekly injection of pegylated-interferon and daily oral ribavirin (PEG-IFN/RBV). This treatment was limited by both tolerability and efficacy, with only about 50% of patients infected with HCV genotype 1, the most prevalent genotype in Western countries, achieving SVR [3].

The approval in 2011 of two HCV protease inhibitors (PIs), telaprevir and boceprevir, to be used in combination with PEG-IFN/RBV, marked an undisputable milestone for HCV therapy, with the SVR rate in phase 3 clinical trials higher than 70% in HCV genotype 1 treatment-naive patients [4]–[8]. However, the enthusiasm for using these first generation PIs is tempered by their side effects and the emergence of resistance to treatment. A second generation of PIs, presenting better safety and resistance profiles, are now in various stages of clinical development, but their direct and indirect mechanisms of action and *in vivo* antiviral effectiveness remain unclear. Yet such information is critical for combining PIs with other direct-acting antiviral agents (DAAs) that have independent mechanisms of action to yield highly potent drug cocktails.

Mathematical modeling of HCV kinetics has provided valuable insights into the modes of action of PEG-IFN/RBV [9], [10] (also see review in [11], [12]). Nevertheless, models developed for PEG-IFN/RBV therapy may not be useful for understanding the determinants of viral decline during PI therapy since these models do not account for intracellular viral replication, which is the main target of DAAs. Recently, multiscale HCV models have been developed that combine both the intracellular and extracellular viral dynamics [13], . One such model formulated as a system of ordinary differential equations (ODEs) was shown to be able to explain some patterns of viral kinetics observed during PI monotherapy, such as the rapid decline of drug sensitive virus and the rapid emergence of drug resistant virus [13].

In this paper, we use a multiscale model of HCV infection and treatment that includes the age structure of infected cells, as well as the dynamics of intracellular viral replication, in order to understand the effects of PI therapy. An approximation of this model was applied to understanding the kinetics of viral decline observed during the first two days following one dose of daclatasvir, a DAA that inhibits the HCV NS5A protein [14]. Here we formulate the model in detail, present its mathematical properties, and derive both short-term and long-term analytical approximations of the model under therapy. We then use the long-term approximation to fit viral kinetic data obtained from eight patients treated for two weeks with danoprevir, a potent second generation PI. We provide for the first time an estimate of the *in vivo* antiviral potency of danoprevir in blocking different stages of viral replication, e.g., reducing intracellular viral RNA production, enhancing its degradation, and inhibiting viral assembly or secretion.

## Materials and Methods

### Patient data

The viral kinetic data we analyzed are from eight treatment naive patients who were infected with HCV genotype 1 and treated with danoprevir monotherapy (200 mg tablets three times a day) for 14 days [15]. Viral loads were measured post treatment initiation at hours 0 (baseline), 2, 4, 6, 8, 12, 16, 24 (day 1), 26, 28, 30, 48 (day 2), 52, 144 (day 6), 148, 192 (day 8), 196, 312 (day 13). The NS3 protease sequence was evaluated by population sequencing at days 0, 2, 6 and 13. Three patients 01-94AB, 03-94EZ, and 03-94SN were found to develop mutations conferring drug resistance, all of them at position R155K. The models we use to analyze the data assume that drug effectiveness is constant over the period of treatment. Hence these models are not suitable for analyzing patient data once drug resistance is apparent and we thus fit the data from these three patients until the time at which resistance was identified. As resistance data were missing at both days 2 and 6 in patients 01-94AB and 03-94SN, viral load data were fit until a rebound was observed at day 6 in 01-94AB and at day 2 in 03-94SN. One patient (04-94XD) had a viral load under the limit of quantification (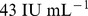 [15]) at day 14, and the data fit was done by setting this viral load to half of the limit of quantification. Data were fitted using both a standard biphasic model and a long-term approximation of the multiscale model developed in this study. Because drugs do not act instantaneously, we assumed the viral load began to decline a short time, 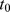, after the onset of therapy.

### The standard biphasic model

The Neumann et al. model [9] has been extensively used to study HCV kinetics during treatment [16]–[18] (see review in [11], [12]). In this model, target cells, 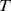, are produced at a rate constant 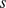, die at per capita rate 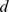, and are infected by virus, 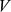, at rate 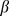. Target cells are assumed to be equivalent and equally available to be infected. Infected cells are assumed to die at per capita rate 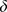. Virions are generated at rate 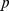 per infected cell, and cleared at rate 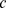 per virion. The model is formulated as the following set of ODEs:
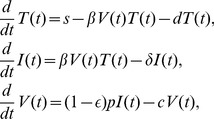
(1)Treatment is assumed to reduce the average viral production rate per infected cell from 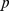 to 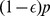, where 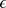 is the *in vivo* antiviral effectiveness of therapy (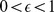). If short-term data after treatment initiation are analyzed, it is often assumed that target cells remain at their pre-therapy level. As a consequence, the ODE system can be linearized and solved. Doing so, one finds
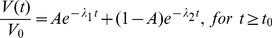
(2)where 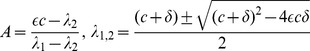, 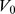 is the baseline viral load before therapy, and 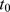 is the pharmacological delay. This model predicts that viral load declines in a biphasic manner, where a short but rapid first phase is followed by a persistent but slower second phase. If 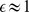, then 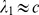 and 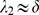. A limitation of this model is that it does not account for the specific stages of the HCV intracellular replication cycle that are targeted by different classes of DAAs and the possibly different effects they exert on the kinetics of viral decline.

### A multiscale model

We extend the biphasic model by including the dynamics of intracellular viral RNA (vRNA). Let 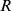 be the quantity of intracellular genomic (i.e. positive-strand) vRNA present in an infected cell. The dynamics of 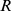 depend on the tradeoff between vRNA production and loss due to degradation and assembly/secretion as virions and can be described by the following equation
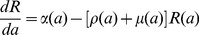
(3)where 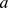 is the age of infection, i.e., the time that has elapsed since an HCV virion has entered the cell. The parameters 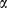, 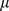 and 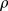 are the age-dependent rates of vRNA production, degradation and assembly/secretion, respectively. For simplicity we do not distinguish between vRNA being packaged into a virion and the virion being secreted. Once vRNA is packaged it is no longer available for replication or degradation, and we assume the packaged virion is secreted. A more complex model would distinguish these processes but at the expense of additional parameters. We assume that a cell is infected by a single virion and hence there is only one vRNA in an infected cell at age 0, i.e., 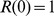. A model similar to Eq. (3) but with constant parameters has been successfully used to fit intracellular vRNA levels in an in vitro replicon system [19], giving us confidence that a simple model can capture many of the major events in vRNA replication. More complex models exist (e.g., Dahari et al. [20] in which the model has 9 equations and 18 parameters) but because they involve many parameters whose values are not known as well as many other intracellular molecules they are not well suited for our purpose here of understanding the major effects of PI therapy.

Combining the equations governing the vRNA kinetics and the cell infection dynamics given by Eq. (1), an age-structured multiscale model of HCV kinetics results that can be described by the following partial differential equations (PDE):
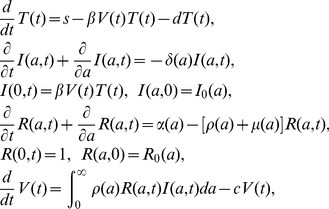
(4)The initial distributions of infected cells and intracellular vRNAs are assumed to be 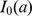 and 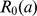, respectively. If 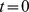 is chosen to be the time of initial infection, then 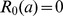. The 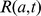 equation would become an ODE (Eq. 3) if 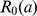 were the steady state distribution since no further time evolution would occur.

Unlike the standard biphasic model, three different antiviral effects of therapy with DAAs can be distinguished in the multiscale model, namely blocking vRNA production (i.e., reducing 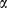 by a factor 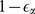), reducing assembly/secretion of virus (i.e., reducing 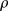 by a factor 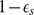), and enhancing the rate of vRNA degradation (i.e., increasing 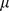 by a factor 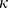), where 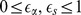 and 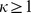 are the effectivenesses of therapy in affecting different processes in the viral life cycle. The full model combining both intra and extracellular viral kinetics under therapy is
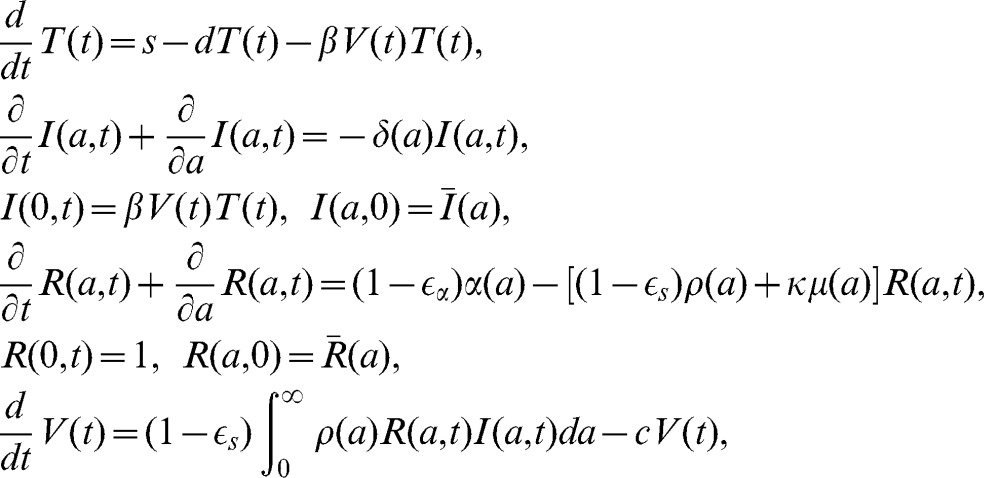
(5)where 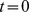 is the time at which treatment is initiated, 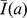 and 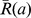 are the steady state distribution of infected cells and intracellular vRNAs, respectively, before therapy, which will be calculated in Results. An additional potential antiviral effect of the therapy on intracellular replication templates will be incorporated into the model later (see the subsection of Long-term Approximation).

## Results

### Analysis of viral decline data using the standard biphasic model

We first fit the patient data using the standard biphasic model (Eq. 2). There are 5 parameters in the prediction of viral decline, including the baseline viral load 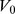 and the pharmacological delay 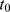. Because of the lack of frequent sampling in the first several hours after treatment initiation, 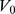 and 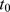 could not be estimated precisely. Thus, we fixed 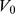 to the last observed viral load before continuous viral reduction was observed, and 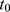 was defined as the mean between the time when 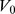 was measured and the time of the next observed data point. For instance, with the sampling of this study, if the last viral load measurement before the viral decline was at 2 hours, and the next viral load was taken at 4 hours, then 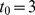 hours. An alternative would be to fix 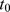 to the time for danoprevir to reach its maximum serum concentration, but as liver concentrations differ we prefer the method given above. The other 3 parameters, 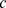, 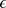, and 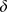 were estimated by non-linear least squares regression using the Levenberg-Marquardt algorithm [21].

**Figure 1:**
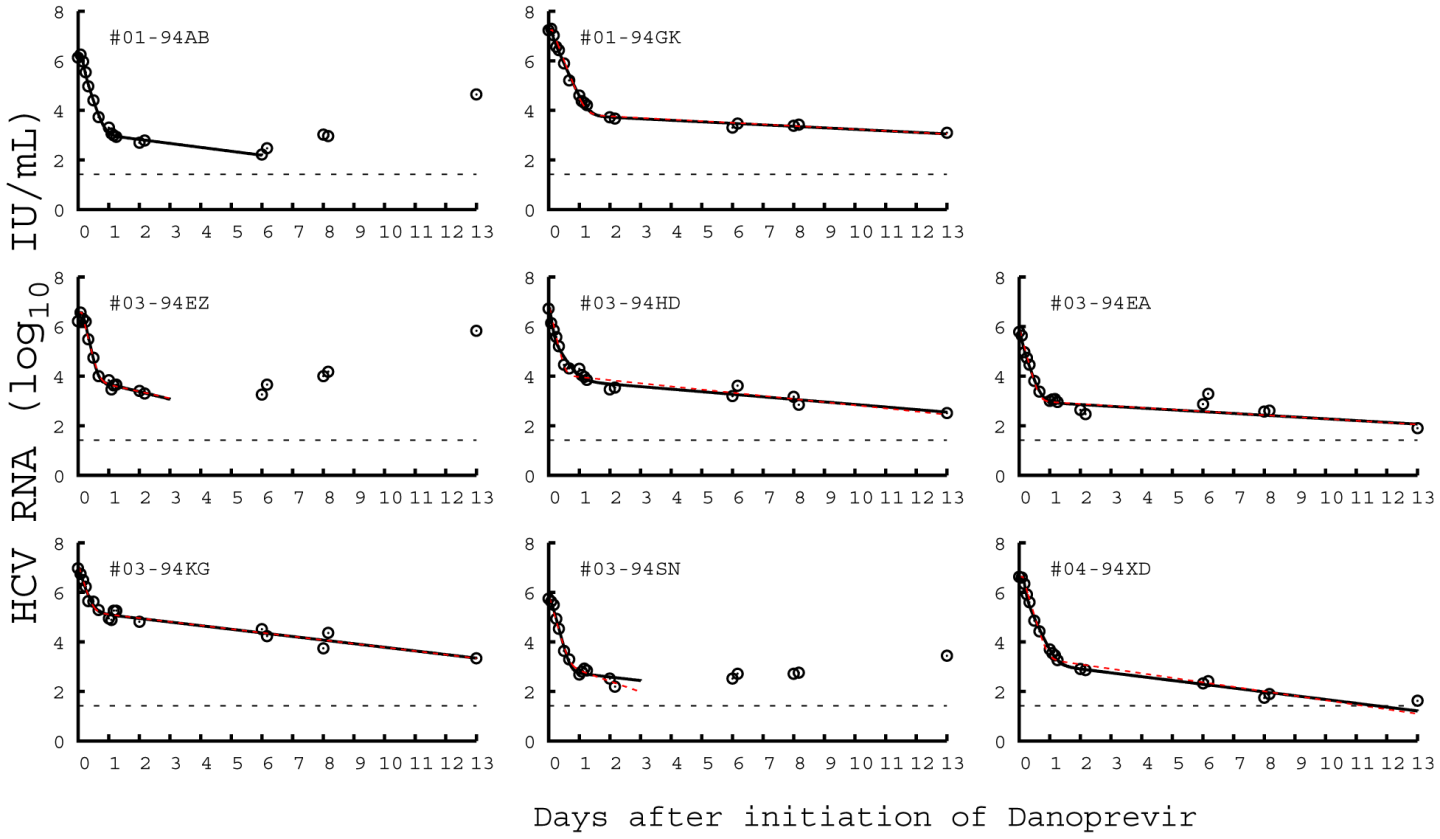
Comparison of viral load data with model predictions for each patient. The prediction from the standard biphasic model is shown by the red dashed line and the prediction from the long-term approximation of the full multiscale model is shown by the black solid line. In most cases the two predicted viral load decay curves overlap and cannot be distinguished. The parameter values used to generate the theoretical curves are the best fit values given in Table 1 and 2, respectively. Viral rebounds occur due to drug resistance and the decay data (circles) was only fit until resistance was detected or rebound was observed. The limit of viral load detection is indicated by the black dashed line.

The biphasic model provides good fits to patient data. The best fits are shown in Figure 1 (red dashed line). Estimates of parameter values on the basis of the best fits are given in Table 1. The biphasic model predicts that danoprevir blocks viral production with mean effectiveness 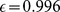. For this model, the slopes of the first-phase and second-phase viral decline reflect the viral clearance rate, 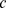, and the death rate of infected cells, 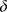, respectively. The average estimates of 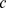 and 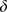 are 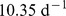 and 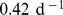, respectively (Table 1). These values are substantially greater than what was typically found during IFN-based therapy [9], where 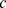 and 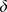 are in the order of 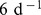 and 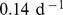, respectively. Interestingly, in another study [14] in which the biphasic model was used to analyze HCV viral decline in patients receiving the HCV NS5A inhibitor daclatasvir, the average estimates of 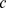 and 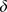 were 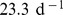 and 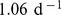, respectively, which are also significantly higher than estimates during IFN-based therapy. It is unlikely that the administration of DAAs such as danoprevir or daclatasvir will enhance the clearance rate of virus, although they could lead to higher estimates of 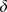 by causing the loss of intracellular viral RNA [13] and ultimately the “cure” of infected cells as has been seen in vitro [22], [23]. To model this effect, as well as to explain the higher value of 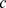 seen with DAAs, we introduce a multiscale model (Eq. 5) that accounts for the different stages of intracellular viral replication that are specifically targeted by DAAs.

**Table 1:** Parameter values with standard errors in parenthesis estimated by fitting the standard biphasic model to viral load data.

Patient	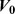 ( 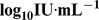 )	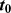 (days)	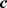 ( 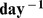 )	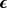	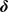 ( 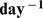 )
01-94AB	6.24	0.12	11.50 (0.80)	0.999 (0.000066)	0.42 (0.060)
01-94GK	7.24	0.12	7.38 (0.33)	0.9995 (0.000071)	0.15 (0.019)
03-94EA	5.79	0.04	10.50 (0.72)	0.998 (0.00022)	0.17 (0.037)
03-94EZ	6.56	0.12	11.35 (0.76)	0.998 (0.00060)	0.65 (0.35)
03-94HD	6.72	0.04	12.44 (1.07)	0.998 (0.00036)	0.29 (0.038)
03-94KG	6.98	0.04	9.40 (1.16)	0.98 (0.0034)	0.35 (0.035)
03-94SN	5.74	0.04	10.03 (0.61)	0.997 (0.00090)	1.0 (0.32)
04-94XD	6.63	0.12	10.26 (0.69)	0.9995 (0.00063)	0.33 (0.040)
**Mean**	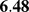	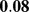	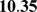 1	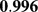	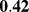 2
**SD**	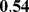	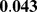	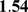	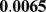	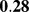

1 Corresponding to a half-life *t*_1/2_ = 0.067 days.

2 Corresponding to a half-life *t*_1/2_ = 1.65 days.

### Analysis of the multiscale model

We analyze the multiscale model and derive analytical approximations that will be used to fit patient data. We begin with the infected steady state of the pre-therapy model (4). For the pre-therapy model, we note that 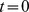 can be any time and does not have to be the time of initial infection. For example, 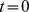 could be the time one starts observing an infected patient. If the patient has not been infected for too long then the steady state has not been reached. A full analysis is needed to determine if the solution of the system will converge to the steady state.

Let
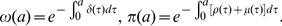
(6)Then 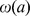 and 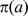 can be interpreted as the probability of an infected cell and an intracellular vRNA surviving to age 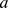, respectively. At steady state, the density of infected cells that have an age 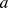 is
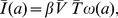
(7)where 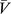 and 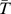 are the steady-state viral load and target cells, respectively. The steady-state level of vRNA within an infected cell of age 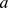 is
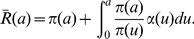
(8)When 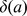, 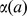, 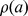, and 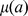 are all constants, we have 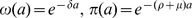, and
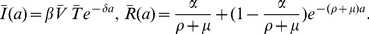
(9)Plugging 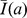 and 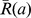 into the 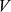 equation in (4), we have
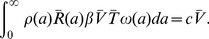
(10)Let
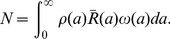
(11)Thus, 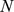 gives the total number of virions produced by one infected cell over its lifespan. This number is usually called the viral burst size [24], [25]. Solving Eq. (11), we obtain 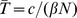. From the first equation of (4), we obtain the steady-state viral load, 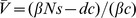. Substituting 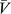 and 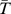 into Eq. (10), we obtain 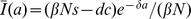. Let 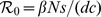. 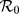 is the basic reproductive ratio of model (4). The infected steady state (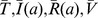) of model (4) is feasible, i.e. has all variables positive, if and only if 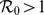. In Text S1, we further show that the infection-free steady state is locally asymptotically stable when 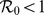 and unstable when 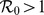, and that the infected steady state is locally asymptotically stable whenever it exists, i.e., when 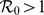.

### Approximation solutions of the multiscale model under therapy

We assume that 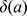, 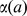, 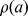, and 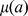 are all constants. Using the method of characteristics one can then show that (see Text S1) after initiation of therapy at time 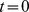, 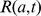 and 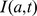 are
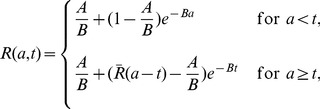
(12)

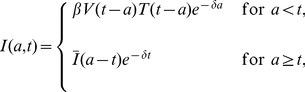
(13)where
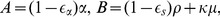
(14)and 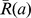 and 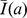 are the steady-state distributions, given in Eq. (9). Even though the distributions of 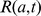 and 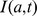 have an analytical form, 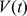 does not. Consequently, the implementation of the full multiscale PDE model and its use for fitting viral load data involve cumbersome calculations. Therefore, it is important to study whether reasonable assumptions can be made to derive relevant analytical approximations of this model in the context of DAA-based therapy.

#### Short-term approximation

We first approximate the viral load decline by assuming that after therapy is initiated infected cells remain at their steady-state distribution, i.e., 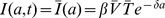. This is equivalent to assuming that new infections (corresponding to the case of 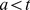) after treatment initiation still occur at the same rate as before treatment. This assumption is reasonable only for a short time after therapy initiation because the rate of new infections will decline in the presence of effective treatment. With this assumption, the total number of infected cells is 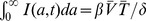, which is exactly the number of infected cells before therapy in the standard biphasic model (2). Assuming that infected cells remain at their pre-therapy level was also used in [9] to study the short-term viral decline under IFN therapy.

Using 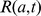 in Eq. (12), we solve the 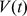 equation in model (5) and obtain (see Text S1)
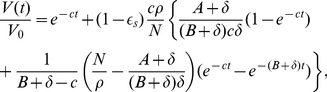
(15)where
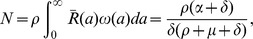


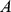
 and 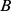 are given in (14), and 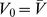 is the baseline viral load before the onset of therapy. Because the assumption of a constant rate of new infection is reasonable only for a short period after therapy initiation, we call Eq. (15) a short-term approximation of the viral decline after therapy.

#### Long-term approximation

Alternatively, we can approximate the viral load decline by neglecting all new infections after the onset of therapy, i.e. assuming 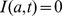 for 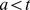. This is reasonable if therapy is potent enough so that viral levels decline profoundly and continuously after therapy is initiated. From Eq. (12), 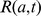 converges to a non-zero steady state solution 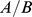. However, this may not be realistic as *in vitro* cell culture systems have shown that under potent therapy vRNA declines continuously and that a complete eradication of vRNA can be obtained after weeks of treatment [22], [23]. Consequently, to study long-term therapy we modify the equation of 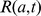 by introducing a new term, 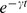, which represents the decay of replication templates (e.g. replication complexes or negative strand HCV RNA) under therapy. Such an exponential term was also used in a model in [19] to fit intracellular vRNA levels in an in vitro replicon system. A more complete model would include another intracellular equation for replication complexes (such as the equation in [13]) but would involve more unknown parameters (see Discussion). In fact, we show in Text S1 that the inclusion of 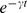 in the 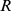 equation is consistent with the formulation of the intracellular model in [13] that explicitly includes the dynamics of replication templates.

With an exponential decay in vRNA production during treatment, the 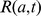 equation becomes
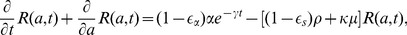
(16)with the initial condition 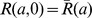 given in (9).

Using this new equation for 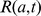 and neglecting all new infections after the onset of therapy, we obtain (see Text S1)
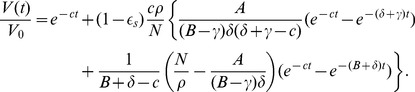
(17)In this approximation, we neglect all new infections during therapy. This is reasonable after therapy has substantially reduced the viral load. Thus, we call Eq. (17) a long-term approximation of the viral decline after therapy.

#### Numerical comparisons

**Figure 2:**
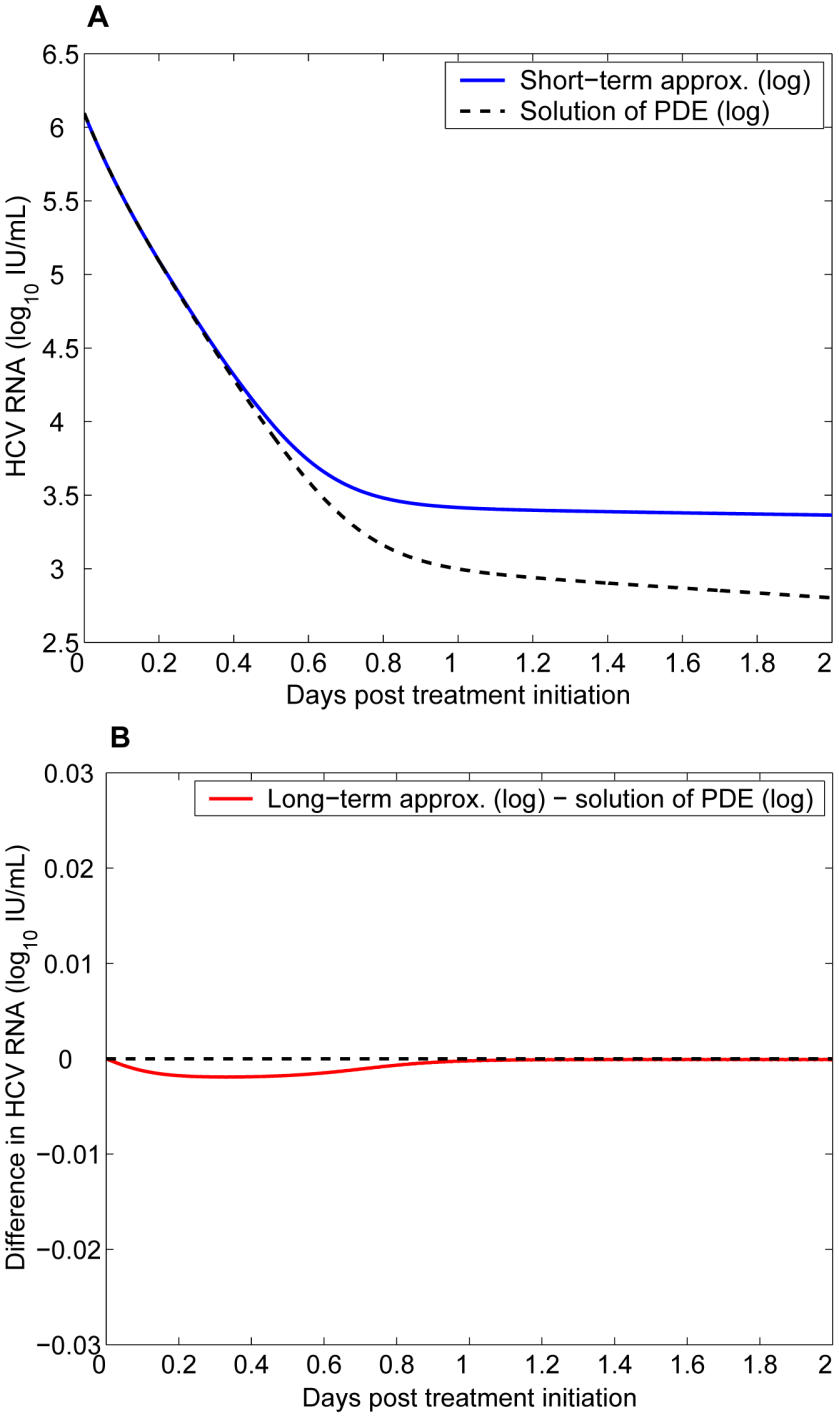
The approximate and the numerical solutions of the multiscale model. **A.** The short-term approximation (blue solid) is compared with the solution of the multiscale PDE model (black dashed). **B.** Difference between the long-term approximation and the solution of the multiscale PDE model. Parameter values, chosen from Table 2 and [30], are 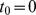, 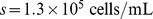, 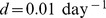, 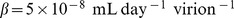, 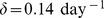, 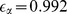, 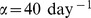, 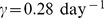, 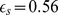, 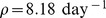, 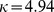, 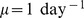, and 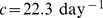.

Under effective drug therapy, the viral load decreases and the rate of new infections, 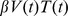, also decreases. Thus, we have 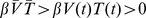. From the assumptions used to derive approximations, we expect that 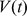 given by the short-term approximation is greater than the prediction of the PDE model, which in turn is greater than the long-term approximation. Numerical results confirm these predictions. In Figure 2A, we show that the short-term approximation agrees well with the solution of the PDE model during the early stage of therapy. However, the short-term approximation approaches a steady state quickly, which is greater than the solution of the multiscale PDE model. In Figure 2B, we compare the long-term approximation with the numerical solution of the PDE model. The long-term approximation is an underestimate of the PDE solution. However, in the parameter range of interest the difference between them is extremely small and the long-term approximation converges to the PDE solution quickly (Figure 2B).

#### Duration of the phases of viral decline

The presence of three exponential terms in the long-term approximation Eq. (17) implies that the multiscale model, unlike the standard biphasic model, may have as many as three phases of viral load decline during therapy. The first exponential term represents the clearance of virus from the circulation (with rate 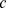). The other two terms represent loss of the material needed to make new virus through a combination of processes. The second exponential term represents the loss of intracellular vRNA by export and degradation as well as the elimination of infected cells (with rate 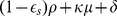), while the third term represents the reduction in vRNA due to loss of replication templates in infected cells as well as the elimination of infected cells (with combined rate 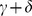).

**Figure 3:**
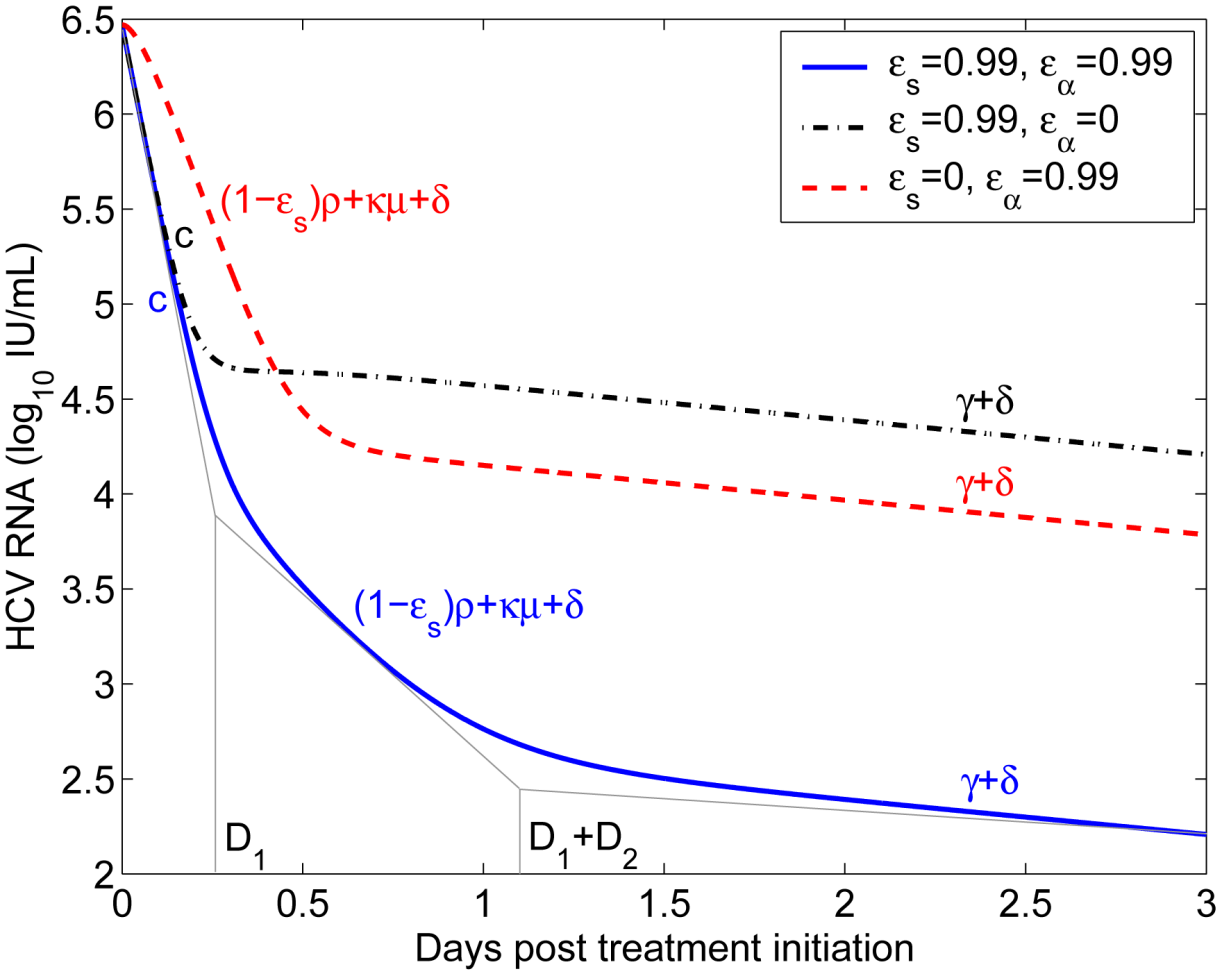
Phases of viral decline affected by the effectiveness of therapy in blocking intracellular viral production and assembly/secretion. When therapy significantly blocks both intracellular viral production (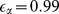) and assembly/secretion (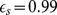), the viral load decline has three phases (blue solid), with slopes 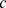, 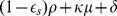, and 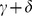, respectively. The duration of the first phase (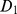) is about 0.25 days and the duration of the second phase (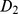) is about 0.88 days using the parameter values below. When 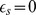, the first-phase viral decline with the slope 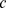 is not visible (red dashed). When 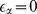, the second-phase viral decline is not visible (black dash-dotted). Parameter values 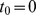, 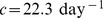, 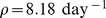, 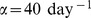, 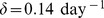, 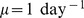, 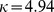, 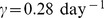, and 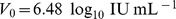 are from Table 2. Because 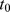 was chosen to be 0, when comparing the predicted duration of the first phase in this figure with clinical data one may want to add the length of the pharmacological delay to 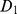.

Three exponential terms do not mean that a triphasic viral decline will always be observed during therapy. In fact, we show in Text S1 that the rapid first phase of viral decline with rate 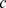 will be observed only if therapy efficiently blocks viral assembly or secretion, i.e. if 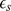 is close to 1. Moreover, in this case we calculated that the first-phase viral decline may not last more than 8 hours based on parameter values estimated from data fitting. Therefore, the rapid first-phase viral decline may not be identified from clinical data if viral load measurements are not taken very frequently after the initiation of treatment. We show in Text S1 that there will not be a visible second-phase viral decline (with the slope 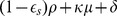) unless 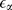, the effectiveness of therapy in blocking intracellular viral production, is close to 1. Numerical simulations with different combinations of 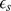 and 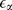 confirm these predictions (Figure 3). In Text S1, we also show the effect of 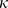 on the viral load decline. Changing 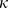 only affects the phase of viral decline with slope 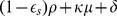.

### Analysis of viral decline data using the multiscale model

We analyzed patient data using the long-term approximation of the multiscale model under therapy. The long-term approximation (Eq. 17) has 11 parameters, including the baseline viral load 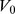 and the pharmacological delay 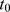. We chose 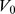 and 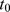 to have the same values as determined for the biphasic model (Table 1). Because parameters that are not related to treatment should not depend upon the DAA being used, we chose 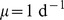, 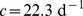 (corresponding to a half-life 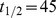 minutes), 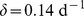 (corresponding to 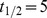 days), 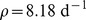 and 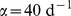 (Table 2), which are consistent with what was estimated *in vitro* [26] and also in patients treated with daclatasvir [14] and IFN [9]. The remaining parameters were the four treatment effectiveness parameters 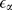, 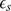, 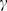 and 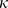 and these were estimated using the same method as used for the biphasic model. Note that changing 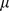 will affect 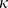 since only 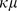 can be estimated. Similarly, changing 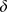 will mainly affect 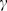 because 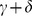 is estimated from the slope of the last phase of viral decline (Figure 3).

**Table 2:** Parameter values with standard errors in parenthesis estimated by fitting the long-term approximation to viral load data and assuming 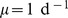, 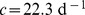, 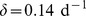, 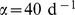 and 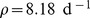.

Patient	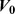 ( 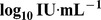 )	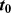 (days)	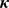	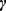 ( 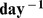 )	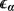	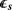
01-94AB	6.24	0.12	6.64 (0.50)	0.23 (0.056)	0.997 (0.00066)	0.78 (0.060)
01-94GK	7.24	0.12	3.52 (0.22)	−0.014^1^ (0.022)	0.999 (0.00015)	0.69 (0.051)
03-94EA	5.79	0.04	5.97 (1.18)	0.0003 (0.047)	0.994 (0.0025)	0.71 (0.18)
03-94EZ	6.56	0.12	6.24 (1.64)	0.55 (0.32)	0.996 (0.0020)	0.13 (0.34)
03-94HD	6.72	0.04	4.07 (0.93)	0.095 (0.048)	0.99 (0.0040)	0.92 (0.036)
03-94KG	6.98	0.04	4.19 (1.46)	0.20 (0.036)	0.97 (0.012)	0.55 (0.25)
03-94SN	5.74	0.04	4.10 (0.72)	0.96 (0.23)	0.995 (0.0015)	0.00 (NA)
04-94XD	6.63	0.12	4.81 (0.22)	0.22 (0.026)	0.999 (0.00030)	0.80 (0.042)
**Mean**	**6.48**	**0.08**	**4.94**	**0.28**	**0.992**	**0.56**
**SD**	**0.54**	**0.043**	**1.18**	**0.33**	**0.0096**	**0.33**

1 This value is not significantly smaller than 0.

The long-term approximation of the multiscale model provides good fits to the viral load data. The best fits are shown in Figure 1 for comparison with the prediction of the biphasic model. Parameter estimates on the basis of the best fits are presented in Table 2. The model predicts that danoprevir significantly reduces vRNA production within infected cells, with mean effectiveness 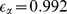, and has a modest effect in blocking viral assembly or secretion, with mean 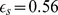. Finding an effect of danoprevir on virion assembly or secretion is consistent with a recent finding that the HCV protease domain is important in late steps in the viral life cycle that involve intracellular assembly of virus [27]. With a modest drug efficacy 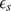, the rapid viral decline with slope 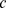 occurs over a very short interval and is not visible in the data from these patients (see Text S1). Because 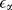 is close to 1, the viral decline with slope 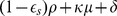 is visible. Thus, the viral load decline observed during the first two days of treatment mainly reflects a combined effect of danoprevir in reducing viral secretion and in enhancing the degradation of vRNA, with mean 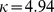 (Table 2).

The viral load decline after day 2 is due to a combined effect of the loss of infected cells, with rate 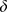, and the exponential reduction in vRNA production, with rate 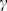 (see Eq. 23). We fixed 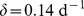, which is the mean value estimated during IFN-based therapy [9], based on the idea that this value represents the rate of immune-mediated death of infected cells, whereas 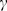 represents the rate of “cure” of infected cells due to the loss of replication complexes. Phenomenologically, 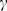 represents the enhancement in the rate of viral decline after day 2 compared to what was observed during IFN-based therapy. Interestingly, we estimated that the mean of 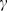 was 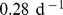, so that the mean long-term viral decline during treatment with danoprevir, 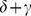, was roughly 3 times more rapid than during IFN therapy. Interestingly, this 3-fold more rapid decline is consistent with the results obtained with the biphasic model where 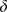 alone represents the rate of long-term decline. In this case, the mean value of 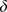 for danoprevir was 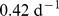 (Table 1), i.e., 3 times greater than 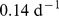, the 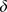 for IFN.

The estimate of 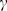 varies among patients. In three patients (01-94GK, 03-94EA, and 03-94HD), the estimates of 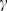 were not significantly different from 0. In two patients (03-94EZ and 03-94SN), the estimates of 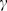 were large (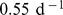 and 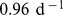, respectively). However, only data until day 2 were used in these two patients, due to viral rebound (see Materials and Methods), which inflates the uncertainty in their parameter estimates. In the other two patients (03-94KG and 04-94XD), although no resistance was detected, the viral decline between day 2 and day 14 was modest. In the patients with 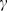 not significantly different from 0, either the viral decline was not enhanced by eradication of viral replicative intermediates in infected cells or the default value chosen for 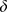 was too high, masking any potential effect of intracellular loss of viral replicative intermediates as our model fits can only accurately estimate 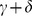 (Figure 3).

We also tested the sensitivity of our estimates to changes in the values of 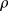 and 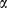. We refitted the long-term approximation to the viral load data assuming 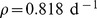 (10-fold smaller than used in previous fitting) and 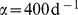 (10-fold larger than used in previous fitting), i.e., assuming the pre-therapy steady state vRNA (given by 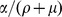) and viral production (given by 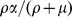) are 50 and 5-fold higher, respectively, than used in the previous fittings. Although the viral load predictions were similar (not shown), some parameter estimates were different (Table 3). Specifically, the estimates of 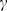 and 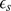 remained nearly unchanged. The mean estimate of 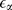, 0.97, was slightly smaller than the previous mean estimate, 0.992. The mean estimate of 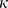, 8.06, was higher than the previous mean estimate, 4.94. One can explain the discrepancy in the estimate of 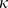 as follows. Because 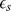 is modest and 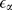 is close to 1, the rapid viral decline with slope 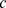 is very short and the first visible phase of viral decline is essentially determined by 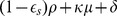. Thus, 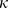 needs to be higher to compensate for the effect of assuming a 10-fold smaller rate of viral secretion, 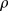.

**Table 3:** Parameter values with standard errors in parenthesis estimated by fitting the long-term approximation to viral load data and assuming 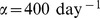, 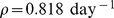, and other fixed parameters as given in Table 2 caption.

Patient	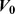 ( 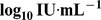 )	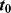 (days)	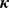	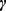 ( 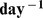 )	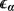	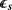
01-94AB	6.24	0.12	8.27 (0.82)	0.23 (0.056)	0.99 (0.0031)	0.78 (0.060)
01-94GK	7.24	0.12	5.70 (0.27)	−0.014^1^ (0.026)	0.997 (0.00071)	0.69 (0.051)
03-94EA	5.79	0.04	8.11 (2.02)	0.00033 (0.047)	0.97 (0.012)	0.71 (0.18)
03-94EZ	6.56	0.12	12.67 (1.82)	0.55 (0.32)	0.98 (0.0088)	0.13 (0.35)
03-94HD	6.72	0.04	4.63 (1.14)	0.095 (0.048)	0.96 (0.019)	0.92 (0.037)
03-94KG	6.98	0.04	7.49 (2.57)	0.19 (0.036)	0.85 (0.060)	0.55 (0.25)
03-94SN	5.74	0.04	11.45 (0.72)	0.96 (0.23)	0.98 (0.0072)	0.00 (NA)
04-94XD	6.63	0.12	6.21 (0.37)	0.22 (0.026)	0.994 (0.0015)	0.81 (0.042)
**Mean**	**6.48**	**0.08**	**8.06**	**0.28**	**0.97**	**0.57**
**SD**	**0.54**	**0.043**	**2.77**	**0.32**	**0.048**	**0.33**

1 This value is not significantly smaller than 0.

## Discussion

Direct-acting antiviral agents that interfere with various intracellular molecular processes in the HCV life cycle are revolutionizing therapy for patients chronically infected with HCV [28], [29]. Two protease inhibitors, telaprevir and boceprevir have been approved by the US Food and Drug Administration to treat HCV infection when used in combination with PEG-IFN/RBV. They can effectively block the NS3-4A protease-dependent cleavage of the HCV polyprotein, which is essential for viral replication. The addition of either of them to therapy with PEG-IFN/RBV has significantly increased the rate of SVR [4]–[8]. These compounds, viewed as the first generation of PIs, have several shortcomings: side effects including serious rash for telaprevir, the necessity to be taken three times a day, and limited effectiveness for non-genotype 1 patients. Moreover, these drugs have a poor resistance profile and drug resistant virus rapidly emerges when these PIs are used as monotherapy [30]. Although still in clinical development there are good expectations that the second generation of PIs will overcome some of these shortcomings (see reviews in [31], [32]). Danoprevir is one such compound and it has shown potent antiviral activities *in vitro*, in the HCV replicon model, as well as in treatment-naive and treatment-experienced patients in combination with PEG-IFN/RBV [15], [33], [34] or mericitabine, an HCV polymerase inhibitor [35]. Despite robust antiviral responses of danoprevir and other DAAs, their mechanisms of action and *in vivo* antiviral efficacy remain unclear.

The standard biphasic viral dynamic model is commonly used to study HCV dynamics in patients on therapy and to estimate the values of parameters such as the virion half-life, the productively infected cell loss rate, and the effectiveness of therapy in blocking viral production. By fitting this model to the viral load decline in HCV patients receiving high daily doses of IFN, Neumann et al. [9] estimated that the viral clearance rate, 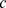, was about 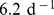, corresponding to a serum half-life, 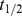, of 2.7 hours. The model was also used to fit the viral load data in patients treated with DAAs [18]. In patients receiving the HCV NS5A inhibitor daclatasvir, the estimate of 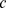 was 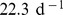, corresponding to a 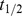 of 45 minutes [14]. Using this model to fit viral load decline from eight patients during two weeks of danoprevir monotherapy, we estimated a mean 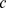 of 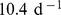 corresponding to a 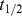 of 1.6 hours. Because the viral clearance rate is a physiological parameter, it should not depend on the specific antiviral agent used in a study. An obvious discrepancy in the estimates of 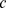 suggests that the biphasic model, which does not include the dynamics of intracellular viral replication within infected cells, may not be optimal for analyzing data in patients treated with DAAs.

In this paper, we introduced a more sophisticated multiscale model including intracellular viral replication that can be used to study the viral kinetic changes in patients treated with DAAs. Unlike the standard biphasic model that only considers the effect of treatment in reducing the average viral production/release per infected cell [9], the multiscale model allows one to estimate three specific effects of the therapy, namely inhibition of vRNA production, enhancement of vRNA degradation, and inhibition of viral assembly and/or secretion.

Analysis of the multiscale model (Text S1 and Figure 3) shows that the first phase of viral decline occurring during the first 6 to 8 hrs after therapy initiation and representing virion clearance in serum can be observed only if therapy substantially blocks viral assembly/secretion. If therapy can also efficiently inhibit intracellular viral production, there is a visible second-phase viral decline, mainly reflecting the loss of material available for producing new virions. This new understanding of the origin of viral decline can be used to explore the mechanisms of action of DAAs. For example, by fitting the long-term approximation of this model to the first two days of viral load data in patients treated with the NS5A inhibitor daclatasvir, it was predicted that daclatasvir efficiently blocks both intracellular viral RNA production and virion assembly/secretion [14]. Thus, during therapy with daclatasvir, the first-phase viral decline reveals the information on the virion clearance rate in serum and the mean half-life of HCV RNA in serum was estimated to be about 45 minutes. This estimate is approximately 4 times shorter than previous estimates made during IFN-based therapy [9], [36]. However, it agrees with the estimates made during the anhepatic phase and immediately after graft reperfusion in the majority of patients who underwent liver transplantation [37], [38]. Also, the estimated virion half-life of 45 minutes can be obtained without the use of the multiscale model by simply estimating via linear regression the rate of viral decline plotted in Figure 4 of ref. [39].

When therapy only moderately blocks viral assembly/secretion, as shown with danoprevir (Table 2) or IFN [14], there is continued packing of vRNAs, made in infected cells before therapy began, and continued release of virions during therapy, which masks the intrinsic virion clearance rate in serum. In this case, the rapid exponential decline of virus reflecting clearance at rate 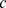 is not observed, and the first visible phase of viral decline mainly reflects the loss rate of vRNA within an infected cell. Here we estimate that danoprevir when given as 200 mg three times a day leads to an enhancement of the vRNA degradation rate by a mean factor of approximately 5. When using the standard biphasic model rather than the multiscale model, this early viral decline phase was attributed to a high viral clearance rate, 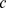, of 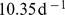. This is substantially higher than the typical value of 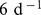 estimated during IFN-based therapy [9]. As our new model indicates that this phase mainly reflects vRNA degradation rather than viral clearance, these results suggest that danoprevir induces a more profound enhancement of vRNA degradation than IFN. Likewise, using the standard biphasic model to fit the viral decline in patients treated with 1250 mg telaprevir given twice a day, 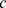 was estimated to be equal to 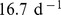 [18]. However, using the new modeling approach, we showed elsewhere that telaprevir, like danoprevir, enhances the vRNA rate of degradation with mean 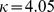 [14]. Direct comparisons of the effects of telprevir and danoprevir should not be made as these results come from different studies, with different patient populations and different doses of drug, but nonetheless these studies suggest both drugs behave similarly.

Why danoprevir and telaprevir enhance the intracellular viral RNA degradation rate remains unclear. It might result from the restoration of cellular antiviral capabilities [40]. Type I IFNs and other inflammatory cytokines can be induced in infected cells that recognize vRNA [41]. However, the HCV NS3-4A protease interferes with this pathway by cleaving the Toll-like receptor 3 adaptor protein TRIF [42], and blocking the activation of IFN regulatory factor 3 (IRF-3), a key cellular antiviral signaling molecule [43]. Thus, inhibition of the NS3-4A protease may simultaneously block viral replication and restore a cellular antiviral response that might promote intracellular viral RNA degradation. One candidate pathway involves ADAR1, an adenosine deaminase that acts on double-stranded RNA. This enzyme is induced by type I IFN, and it specifically eliminates HCV RNA by adenosine to inosine editing [44].

Approximately two days after the start of danoprevir therapy, a subsequent and persistent phase of viral decline was observed that was about three times faster than typically found during IFN-based therapy [9]. Using the standard biphasic model, one would attribute this enhanced phase of viral decline to an elevated loss rate of infected cells. However, it seems unlikely that this elevated loss rate is due to cell death as no increase in the level of alanine aminotransferase (ALT), an enzyme released from damaged or dead hepatocytes, was observed in the 8 patients treated with danoprevir. We hypothesized that the elevated rate of viral decline is due to a continuous reduction in vRNA production, with the rate 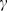. This assumption seems reasonable because HCV negative strand RNAs (or equivalently replication complexes) degrade [20] and are not replaced or inefficiently replaced if RNA replication is largely inhibited by effective therapy. A more complete model would include the dynamics of formation and elimination of replication complexes, as done in [13]. However, we would then have additional intracellular equations and need to determine more unknown parameters. Assuming an exponential decay in the vRNA production rate (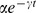) is the simplest way to capture this effect. The same approach was used to study the dynamics of genotype 1b subgenomic replicon RNA under treatment with 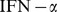 [19].

The long-term approximation in which all new infections are neglected agrees well with the prediction of the full model in the context of an effective therapy. However, the approximation cannot be applied to the full data set from three patients (01-94AB, 03-94EZ, and 03-94SN) in which viral rebound was observed during the 2-week monotherapy. Virologic escape during danoprevir monotherapy was reported to be HCV subtype dependent and mainly due to the emergence of drug resistance associated with the substitution R155K [45]. Further, even in patients with no observed escape resistant variants may be present and slowing viral decline rates. This is probably a common feature of all drugs with a low genetic barrier to resistance. Thus, the current model, assuming no drug resistance, may be underestimating the effect of danoprevir. Extending the model to include two strains [30] or multiple strains [46]–[48] is one method to study the evolution of drug resistance, estimate the *in vivo* fitness of drug-resistant HCV variants, and quantify the effects of resistant variants on the kinetics of HCV RNA decline. In addition, the current model assumes constant drug efficacy. Pharmacokinetic/pharmacodynamic models could be included to describe the time-varying treatment effectiveness [49]–[51]. However, more data are needed for parameter estimation.

In the context of potent therapy leading to a continuous viral decline, mathematical analysis of the standard biphasic model has shown that an additional effect of drug in blocking infection and/or viral entry has no or very limited effect on the kinetics of viral decline [9], [52]. This is the reason why such an effect was not studied here. However, in the context of emerging drug resistant virus or new cell infection, the ability of a drug to block viral entry should be considered as it could have a significant effect in delaying or preventing a viral breakthrough by preventing the ability of resistant virus to infect and propagate in new cells.

Another limitation of our model is that we have assumed that 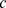 is constant and not influenced by danoprevir. As far as we know there is no evidence that a protease inhibitor such as danoprevir will influence virion clearance. However, if danoprevir did increase 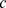 from about the 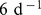 seen with IFN to the 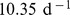 estimated here using the standard model, i.e. by a factor of 2, then the viral load would decline by a factor of 2 during the first phase and not by the more than 2 orders of magnitude observed. Thus, any increase of 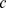 would not explain the profound drop in viral load seen during the first phase. Nonetheless, we cannot rule out the possibility that 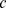 is increased along with the other effects we predicted, such as the block in HCV RNA replication.

In summary, we employed a multiscale model that includes both intracellular RNA replication and extracellular infection dynamics to study the viral load change in HCV patients treated with DAAs. We determined the biological parameters that contribute to different phases of viral decline after initiation of therapy. Applying the model to viral load data from patients treated with a new HCV protease inhibitor, danoprevir, suggests that danoprevir significantly blocks intracellular viral production and enhances viral degradation, while it moderately inhibits viral assembly/secretion. The multiscale model provides a theoretical framework that can be used to explore the mechanisms of action of other DAAs and thus should be useful in furthering drug development for HCV and for optimizing antiviral therapy.

## Supporting Information

Text S1 Model analysis and approximation.
(PDF)
