# Supplementary material for: Analysis of Hepatitis C Virus Decline during Treatment with the Protease Inhibitor Danoprevir Using a Multiscale Model
Source: PLoS Comput Biol. 2013 Mar 14;9(3):e1002959. doi: 10.1371/journal.pcbi.1002959 (PMC3597560; doi:10.1371/journal.pcbi.1002959)
Supplement: Text S1 — Model analysis and approximation. (PDF) [file pcbi.1002959.s001.pdf]

Text S1. Model analysis and approximation

1. Analysis of the pre-therapy model.

We solve the $R(a,t)$ equation in the pre-therapy model. The characteristic curves are $t - a =$ constant. We assume that they intersect with the a-axis at $(a_0, 0)$ and intersect with the t-axis at $(0, t_0)$, where $a_0 \geq 0$ and $t_0 \geq 0$.

When $a \geq t$, the characteristic curves can be described by the parametric equation $t = \tau$, $a = \tau + a_0$, where τ is a free parameter. When τ increases from 0 to t, $a(\tau)$ increases from a_0 to a and $t(\tau)$ increases from 0 to t. Along the characteristic curves, we have

$$\frac{dR(a(\tau), t(\tau))}{d\tau} = \frac{\partial R}{\partial t}\frac{dt}{d\tau} + \frac{\partial R}{\partial a}\frac{da}{d\tau} = \alpha(a(\tau)) - [\rho(a(\tau)) + \mu(a(\tau))]R(a(\tau), t(\tau)).$$

Using the variation of constants formula, we obtain

$$R(a,t) = R(a_0, 0)e^{-\int_0^t [\rho(a(\tau))+\mu(a(\tau))]d\tau} + e^{-\int_0^t [\rho(a(\tau))+\mu(a(\tau))]d\tau} \int_0^t \alpha(a(u))e^{\int_0^u [\rho(a(\tau))+\mu(a(\tau))]d\tau} du.$$

Note that

$$\int_0^t [\rho(a(\tau)) + \mu(a(\tau))]d\tau = \int_{a_0}^a [\rho(\varsigma) + \mu(\varsigma)]d\varsigma,$$

we have

$$e^{-\int_0^t [\rho(a(\tau))+\mu(a(\tau))]d\tau} = e^{-\int_{a_0}^a [\rho(\varsigma)+\mu(\varsigma)]d\varsigma} = \frac{\pi(a)}{\pi(a_0)},$$

where

$$\pi(a) = e^{-\int_0^a [\rho(\tau)+\mu(\tau)]d\tau}.$$

We also have

$$\int_0^t \alpha(a(u))e^{\int_0^u [\rho(a(\tau))+\mu(a(\tau))]d\tau} du = \int_0^t \alpha(a(u))e^{\int_{a_0}^{a_0+u} [\rho(\eta)+\mu(\eta)]d\eta} du$$

$$= \int_{a_0}^a \alpha(\varsigma)e^{\int_{a_0}^{\varsigma} [\rho(\eta)+\mu(\eta)]d\eta} d\varsigma = \int_{a_0}^a \alpha(\varsigma)\frac{\pi(a_0)}{\pi(\varsigma)}d\varsigma.$$

Thus, when $a \geq t$, we obtain

$$R(a,t) = R(a_0, 0)\frac{\pi(a)}{\pi(a_0)} + \int_{a_0}^a \frac{\pi(a)}{\pi(\varsigma)}\alpha(\varsigma)d\varsigma = R_0(a-t)\frac{\pi(a)}{\pi(a-t)} + \int_0^t \frac{\pi(a)}{\pi(a-u)}\alpha(a-u)du.$$

When $a < t$, the characteristic curves can be described by $t = \tau$, $a = \tau - t_0$. When τ increases from t_0 to t, $a(\tau)$ increases from 0 to a and $t(\tau)$ increases from t_0 to t. Using the method of

characteristics again, we obtain

$$R(a,t) = R(0,t-a)\pi(a) + \int_0^a \frac{\pi(a)}{\pi(u)}\alpha(u)du.$$

Thus, we have a complete solution for $R(a,t)$, given by

$$R(a,t) = \begin{cases} \pi(a) + \int_0^a \frac{\pi(a)}{\pi(u)}\alpha(u)du & \textit{for } a < t, \\ \\ R_0(a-t)\frac{\pi(a)}{\pi(a-t)} + \int_0^t \frac{\pi(a)}{\pi(a-u)}\alpha(a-u)du & \textit{for } a \geq t. \end{cases} \tag{1}$$

Similarly, integrating the I equation in the pre-therapy model along the characteristic lines, $t - a =$ constant, we get the solution of $I(a,t)$, given by

$$I(a,t) = \begin{cases} \beta V(t-a)T(t-a)\omega(a) & \textit{for } a < t, \\ \\ I_0(a-t)\frac{\omega(a)}{\omega(a-t)} & \textit{for } a \geq t, \end{cases} \tag{2}$$

where $\omega(a) = e^{-\int_0^a \delta(\tau)d\tau}$.

When $\delta(a)$, $\alpha(a)$, $\rho(a)$, and $\mu(a)$ are all constants, $R(a,t)$ and $I(a,t)$ become

$$R(a,t) = \begin{cases} \frac{\alpha}{\rho+\mu} + (1 - \frac{\alpha}{\rho+\mu})e^{-(\rho+\mu)a} & \textit{for } a < t, \\ \\ \frac{\alpha}{\rho+\mu} + [R_0(a-t) - \frac{\alpha}{\rho+\mu}]e^{-(\rho+\mu)t} & \textit{for } a \geq t, \end{cases} \tag{3}$$

$$I(a,t) = \begin{cases} \beta V(t-a)T(t-a)e^{-\delta a} & \textit{for } a < t, \\ \\ I_0(a-t)e^{-\delta t} & \textit{for } a \geq t. \end{cases} \tag{4}$$

The post-therapy model has an analogous solution where the constants α, ρ, and μ are modulated by the drug effects and become $(1-\epsilon_\alpha)\alpha$, $(1-\epsilon_s)\rho$, and $\kappa\mu$, respectively.

We show that the infection-free steady state is locally asymptotically stable when $\mathcal{R}_0 < 1$ and unstable when $\mathcal{R}_0 < 1$, and that the infected steady state is locally asymptotically stable whenever it exists (i.e. $\mathcal{R}_0 > 1$), where $\mathcal{R}_0 = \beta N s/(dc)$. Analyzing the stability of the steady states of the

pre-therapy model is equivalent to analysis of the following limiting system [1]

$$\begin{aligned} \frac{d}{dt}T(t) &= s - dT(t) - \beta V(t)T(t), \\ \frac{d}{dt}V(t) &= \int_0^\infty \rho(a)\beta V(t-a)T(t-a)\omega(a)\bar{R}(a)da - cV(t), \end{aligned} \tag{5}$$

where $\bar{R}(a)$ is the steady state distribution of intracellular vRNAs, given by

$$\bar{R}(a) = \pi(a) + \int_0^a \frac{\pi(a)}{\pi(u)}\alpha(u)du.$$

The infected and infection-free steady states for the limiting system are $(\bar{T}, \bar{V})$ and $(s/d, 0)$, respectively, with

$$\bar{T} = \frac{c}{\beta N}, \; \bar{V} = \frac{s - d\bar{T}}{\beta\bar{T}} = \frac{sN}{c} - \frac{d}{\beta} = \frac{d}{\beta}(\mathcal{R}_0 - 1) \tag{6}$$

where N is the burst size, given by

$$N = \int_0^\infty \rho(a)\bar{R}(a)\omega(a)da.$$

It is clear that the infected steady state exists if and only if $\mathcal{R}_0 > 1$.

The Jacobian matrix for the limiting system is

$$J = \begin{pmatrix} -d - \beta\bar{V} - \lambda & -\beta\bar{T} \\ \beta\bar{V}\int_0^\infty \rho(a)\omega(a)\bar{R}(a)e^{-\lambda a}da & \beta\bar{T}\int_0^\infty \rho(a)\omega(a)\bar{R}(a)e^{-\lambda a}da - c - \lambda \end{pmatrix} \tag{7}$$

where λ is an eigenvalue.

At the infection-free steady state, the characteristic equation is

$$(\lambda + d)\left[\lambda - \beta\bar{T}\int_0^\infty \rho(a)\omega(a)\bar{R}(a)e^{-\lambda a}da + c\right] = 0. \tag{8}$$

One eigenvalue is $\lambda = -d$ and all other eigenvalues are determined by

$$\lambda - \beta\bar{T}\int_0^\infty \rho(a)\omega(a)\bar{R}(a)e^{-\lambda a}da + c = 0, \tag{9}$$

which can be rewritten as

$$\frac{\lambda}{c} + 1 = \mathcal{R}_0 \frac{\int_0^\infty \rho(a)\omega(a)\bar{R}(a)e^{-\lambda a}da}{\int_0^\infty \rho(a)\omega(a)\bar{R}(a)da}. \tag{10}$$

For all complex roots λ with non-negative real parts,

$$|\int_0^\infty \rho(a)\omega(a)\bar{R}(a)e^{-\lambda a}da| \leq \int_0^\infty \rho(a)\omega(a)\bar{R}(a)da.$$

Thus, the modulus of the right hand side of (10) is less than 1 when $\mathcal{R}_0 < 1$. Because the modulus of the left hand side of (10) is always greater than or equal to 1 for λ with non-negative real parts, we conclude that all roots of the characteristic equation (9) have negative real parts when $\mathcal{R}_0 < 1$. This shows that the infection-free steady state is locally asymptotically stable when $\mathcal{R}_0 < 1$.

When $\mathcal{R}_0 > 1$, we let

$$f(\lambda) = \frac{\lambda}{c} + 1 - \mathcal{R}_0 \frac{\int_0^\infty \rho(a)\omega(a)\bar{R}(a)e^{-\lambda a}da}{\int_0^\infty \rho(a)\omega(a)\bar{R}(a)da}.$$

It is clear that $f(0) = 1 - \mathcal{R}_0 < 0$ and $f(\lambda) \to \infty$ as $\lambda \to \infty$. Thus, there exists a positive root for the equation $f(\lambda) = 0$. This shows that the characteristic equation (9) has at least one positive root. Thus, the infection-free steady state is unstable when $\mathcal{R}_0 > 1$.

At the infected steady state, the characteristic equation is

$$(\lambda + d + \beta\bar{V})\left[\lambda + c - \beta\bar{T}\int_0^\infty \rho(a)\omega(a)\bar{R}(a)e^{-\lambda a}da\right] + \beta\bar{T}\beta\bar{V}\int_0^\infty \rho(a)\omega(a)\bar{R}(a)e^{-\lambda a}da = 0. \quad (11)$$

Considering that $\bar{T} = c/(\beta N)$ and $N = \int_0^\infty \rho(a)\omega(a)\bar{R}(a)da$, Eq. (11) can be rewritten as

$$(\lambda + d + \beta\bar{V})(\frac{\lambda}{c} + 1) = (\lambda + d)\frac{\int_0^\infty \rho(a)\omega(a)\bar{R}(a)e^{-\lambda a}da}{\int_0^\infty \rho(a)\omega(a)\bar{R}(a)da}. \quad (12)$$

For all complex roots λ with non-negative real parts, the modulus of the left hand side of (12) is greater than the modulus of the right hand side. Thus, the characteristic equation (11) has no roots with non-negative real parts. Therefore, the infected steady state is locally asymptotically stable whenever it exists.

2. Short-term approximation of viral load decline after initiation of therapy.

We solve $R(a,t)$ and $I(a,t)$ in the model under therapy. We assume that the system is in the infected steady state at the onset of therapy at a time we call $t = 0$. We also assume that $\delta(a)$, $\alpha(a)$, $\rho(a)$, and $\mu(a)$ are all constants to obtain explicit approximations of the viral load decline during therapy. Similar to Eq. (3) and (4), we obtain a complete solution for $R(a,t)$ and $I(a,t)$ under therapy, given by

$$R(a,t) = \begin{cases} \frac{A}{B} + (1 - \frac{A}{B})e^{-Ba} & \text{for } a < t, \\ \frac{A}{B} + \left(\bar{R}(a-t) - \frac{A}{B}\right)e^{-Bt} & \text{for } a \geq t. \end{cases} \quad (13)$$

$$I(a,t) = \begin{cases} \beta V(t-a)T(t-a)e^{-\delta a} & \textit{for } a < t, \\ \\ \bar{I}(a-t)e^{-\delta t} & \textit{for } a \geq t, \end{cases} \tag{14}$$

where $A = (1-\epsilon_\alpha)\alpha$ and $B = (1-\epsilon_s)\rho + \kappa\mu$. $\bar{R}(a)$ and $\bar{I}(a)$ are the steady state distribution of vRNAs and infected cells, respectively, before the onset of therapy, and are given by

$$\bar{R}(a) = \frac{\alpha}{\rho+\mu} + (1 - \frac{\alpha}{\rho+\mu})e^{-(\rho+\mu)a}, \quad \bar{I}(a) = \beta\bar{V}\bar{T}e^{-\delta a}.$$

Thus, for $a \geq t$, $I(a,t) = \bar{I}(a-t)e^{-\delta t} = \beta\bar{V}\bar{T}e^{-\delta a}$.

We approximate the viral load decline by assuming that after therapy is initiated infected cells remain at their steady state distribution, i.e., $I(a,t) = \bar{I}(a) = \beta\bar{V}\bar{T}e^{-\delta a}$. This is equivalent to assuming that new infections (corresponding to $a < t$) occur at a rate $\beta\bar{V}\bar{T}$ after therapy initiation. This assumption is reasonable only for a short time after therapy initiation because new infections will decline in the presence of effective treatment. In this case, the virus equation becomes

$$\frac{d}{dt}V(t) = (1-\epsilon_s)\rho\int_0^\infty R(a,t)\bar{I}(a)da - cV(t). \tag{15}$$

In consideration of $R(a,t)$ in (13), we split the integral in the above equation into two parts

$$\int_0^\infty R(a,t)\bar{I}(a)da = \int_0^t R(a,t)\bar{I}(a)da + \int_t^\infty R(a,t)\bar{I}(a)da.$$

We calculate the first part and obtain

$$\begin{aligned} \int_0^t R(a,t)\bar{I}(a)da &= \int_0^t \left[\frac{A}{B} + (1-\frac{A}{B})e^{-Ba}\right]\beta\bar{V}\bar{T}e^{-\delta a}da \\ &= \beta\bar{V}\bar{T}\left[\frac{A+\delta}{(B+\delta)\delta} - \frac{A}{B\delta}e^{-\delta t} + \frac{A-B}{(B+\delta)B}e^{-(B+\delta)t}\right]. \end{aligned}$$

Similarly, we calculate the second part and obtain

$$\begin{aligned} \int_t^\infty R(a,t)\bar{I}(a)da &= \int_t^\infty \left[\frac{A}{B} + \left(\frac{\alpha}{\rho+\mu} + (1-\frac{\alpha}{\rho+\mu})e^{-(\rho+\mu)(a-t)} - \frac{A}{B}\right)e^{-Bt}\right]\beta\bar{V}\bar{T}e^{-\delta a}da \\ &= \beta\bar{V}\bar{T}\left[\frac{A}{B\delta}e^{-\delta t}(1-e^{-Bt}) + \frac{N}{\rho}e^{-(B+\delta)t}\right], \end{aligned}$$

where

$$N = \int_0^\infty \rho\bar{R}(a)\omega(a)da = \frac{\rho(\alpha+\delta)}{\delta(\rho+\mu+\delta)}.$$

Adding the above two integrals and simplifying, we have

$$\int_0^\infty R(a,t)\bar{I}(a)da = \frac{c}{N}\bar{V}\left\{\frac{A+\delta}{(B+\delta)\delta} + \left(\frac{N}{\rho} - \frac{A+\delta}{(B+\delta)\delta}\right)e^{-(B+\delta)t}\right\}. \tag{16}$$

Plugging (16) into (15) and solving for $V(t)$, we obtain

$$\frac{V(t)}{V_0} = e^{-ct} + (1-\epsilon_s)\frac{c\rho}{N}\left\{\frac{A+\delta}{(B+\delta)c\delta}(1-e^{-ct}) + \frac{1}{B+\delta-c}\left(\frac{N}{\rho} - \frac{A+\delta}{(B+\delta)\delta}\right)(e^{-ct}-e^{-(B+\delta)t})\right\}, \tag{17}$$

where

$$A = (1-\epsilon_\alpha)\alpha,\ B = (1-\epsilon_s)\rho + \kappa\mu,\ N = \frac{\rho(\alpha+\delta)}{\delta(\rho+\mu+\delta)},$$

and $V_0 = \bar{V}$ is the baseline viral load before the onset of therapy. Because the assumption that new infections occur at a rate $\beta\bar{V}\bar{T}$ is reasonable only for a short time after therapy initiation, we call Eq. (17) a short-term approximation of the viral decline after therapy.

3. Inclusion of $e^{-\gamma t}$ in the R equation to represent the decay of replication templates.

From Eq. (13), $R(a,t)$ will converge to a non-zero steady state solution A/B. However, this is unrealistic under effective therapy since all viral RNA can be eliminated with long-term treatment [2]. Thus, in this case, we modify the equation of $R(a,t)$ by introducing a new term, $e^{-\gamma t}$, which represents the decay of replication templates (e.g. replication complexes or negative strand HCV RNA) under therapy. The $R(a,t)$ equation becomes

$$\frac{\partial}{\partial t}R(a,t) + \frac{\partial}{\partial a}R(a,t) = (1-\epsilon_\alpha)\alpha e^{-\gamma t} - [(1-\epsilon_s)\rho + \kappa\mu]R(a,t), \tag{18}$$

with the initial condition

$$\bar{R}(a) = \frac{\alpha}{\rho+\mu} + (1 - \frac{\alpha}{\rho+\mu})e^{-(\rho+\mu)a}.$$

The inclusion of $e^{-\gamma t}$ in the R equation is consistent with the formulation of the intracellular model that explicitly includes the dynamics of replication complexes in [3]. The intracellular model in [3] is given by

$$\begin{aligned} \frac{d}{dt}U(t) &= \beta_u R(1 - \frac{U}{U_{max}}) - \gamma U, \\ \frac{d}{dt}R(t) &= \alpha U - \rho R - \mu R, \end{aligned} \tag{19}$$

where $U(t)$ is the quantity of HCV replication complexes. Intracellular viral RNA (R) serves as a template for the generation of replication complexes with a maximum rate β_u. U_{max} is the maximum number of replication complexes within a cell. U serves as a template for the generation of α intracellular viral RNAs per replication complex per unit of time. Other parameters are the same as those in this paper.

Similar to Eq. (5) in the main text, we incorporate three possible effects of DAA into the above model. The model becomes

$$\frac{d}{dt}U(t) = \beta_u R(1 - \frac{U}{U_{max}}) - \gamma U, \qquad (20)$$
$$\frac{d}{dt}R(t) = AU - BR,$$

where $A = (1 - \epsilon_\alpha)\alpha$ and $B = (1 - \epsilon_s)\rho + \kappa\mu$.

Under potent therapy, the system will converge to the infection-free steady state $(\bar{U}, \bar{R}) = (0, 0)$. The Jacobian at this steady state is

$$J(0,0) = \begin{pmatrix} -\gamma & \beta_u \\ A & -B \end{pmatrix}$$

The eigenvalues are

$$\lambda_{1,2} = \frac{-(\gamma + B) \pm \sqrt{(\gamma + B)^2 - 4(\gamma B - A\beta_u)}}{2}.$$

Under potent therapy, $4A\beta_u = 4(1-\epsilon_\alpha)\alpha\beta_u << (\gamma - B)^2$. Thus, the eigenvalues are approximately $-\gamma$ and $-B$. Therefore, under potent therapy, the level of intracellular viral RNA will decline in a biphasic manner, with two slopes B and γ. This is consistent with the prediction by the age-structured equation (18) for $R(a,t)$ (see the solution of $R(a,t)$ in (22)).

4. Long-term approximation of viral load decline after initiation of therapy.

We approximate the viral load decline by neglecting all new infections after the onset of therapy, i.e., by assuming $I(a,t) = R(a,t) = 0$ for $a < t$. When $a \geq t$, the characteristic curves of equation (18), $t - a =$ constant, can be described by the parametric equation $t = \tau$, $a = \tau + a_0$. Along the characteristic curves, we have

$$\frac{dR(a(\tau), t(\tau))}{d\tau} = (1 - \epsilon_\alpha)\alpha e^{-\gamma t(\tau)} - [(1 - \epsilon_s)\rho + \kappa\mu]R(a(\tau), t(\tau)).$$

Considering that $A = (1 - \epsilon_\alpha)\alpha$ and $B = (1 - \epsilon_s)\rho + \kappa\mu$, we have

$$\frac{dR}{d\tau} = Ae^{-\gamma\tau} - BR. \qquad (21)$$

Integrating τ from 0 to t, we have

$$\begin{aligned} R(a,t) &= R(a_0, 0)e^{-Bt} + e^{-Bt}\int_0^t e^{Bu}Ae^{-\gamma u}du \\ &= \frac{A}{B-\gamma}e^{-\gamma t} + [\bar{R}(a-t) - \frac{A}{B-\gamma}]e^{-Bt}, \ for \ \ a \geq t. \end{aligned} \qquad (22)$$

The virus equation in the model under therapy becomes

$$\frac{d}{dt}V(t) = (1-\epsilon_s)\rho \int_t^\infty R(a,t)I(a,t)da - cV(t). \tag{23}$$

Using $R(a,t)$ in Eq. (22) and $I(a,t) = \bar{I}(a-t)e^{-\delta t} = \beta\bar{V}\bar{T}e^{-\delta a}$ for $a \geq t$, we calculate the integral

$$\begin{aligned}
\int_t^\infty R(a,t)I(a,t)da &= \int_t^\infty \left\{\frac{A}{B-\gamma}e^{-\gamma t} + [\bar{R}(a-t) - \frac{A}{B-\gamma}]e^{-Bt}\right\}\beta\bar{V}\bar{T}e^{-\delta a}da \\
&= \beta\bar{V}\bar{T}\int_t^\infty \left\{\frac{A}{B-\gamma}e^{-\gamma t} + [\frac{\alpha}{\rho+\mu} + (1-\frac{\alpha}{\rho+\mu})e^{-(\rho+\mu)(a-t)} - \frac{A}{B-\gamma}]e^{-Bt}\right\}e^{-\delta a}da \\
&= \beta\bar{V}\bar{T}\left\{\frac{A}{(B-\gamma)\delta}e^{-(\delta+\gamma)t} + [\frac{\alpha+\delta}{\delta(\rho+\mu+\delta)} - \frac{A}{(B-\gamma)\delta}]e^{-(B+\delta)t}\right\}.
\end{aligned}$$

Considering that

$$N = \frac{\rho(\alpha+\delta)}{\delta(\rho+\mu+\delta)} \quad \text{and} \quad \beta\bar{V}\bar{T} = \frac{c\bar{V}}{N},$$

we have

$$\int_t^\infty R(a,t)I(a,t)da = \frac{c\bar{V}}{N}\left\{\frac{A}{(B-\gamma)\delta}e^{-(\delta+\gamma)t} + [\frac{N}{\rho} - \frac{A}{(B-\gamma)\delta}]e^{-(B+\delta)t}\right\}.$$

Plugging the above integral into the virus equation (23) and solving for $V(t)$, we obtain

$$\begin{aligned}
\frac{V(t)}{V_0} = e^{-ct} + (1-\epsilon_s)\frac{c\rho}{N}\Bigg\{&\frac{A}{(B-\gamma)\delta(\delta+\gamma-c)}(e^{-ct} - e^{-(\delta+\gamma)t}) \\
&+ \frac{1}{B+\delta-c}\left(\frac{N}{\rho} - \frac{A}{(B-\gamma)\delta}\right)(e^{-ct} - e^{-(B+\delta)t})\Bigg\}.
\end{aligned} \tag{24}$$

In this approximation, we neglect all new infections during therapy. This is reasonable after therapy for a period of time that substantially reduces the viral load. Thus, we call Eq. (24) a long-term approximation of the viral decline after therapy.

5. Duration of phases of viral decline.

Because the long-term approximation (24) includes three exponential terms, the viral load decline has three phases under certain conditions. We can approximate the duration of the first and second phases of viral decline under therapy.

Equation (24) can be rewritten as

$$\frac{V(t)}{V_0} = C_1 e^{-ct} + C_2 e^{-(B+\delta)t} + C_3 e^{-(\gamma+\delta)t},$$

where

$$C_1 = 1 - (1-\epsilon_s)\frac{c\rho}{N}\left[\frac{A}{(B-\gamma)\delta(c-\delta-\gamma)} + \frac{1}{c-B-\delta}\left(\frac{N}{\rho} - \frac{A}{(B-\gamma)\delta}\right)\right],$$

$$C_2 = (1-\epsilon_s)\frac{c\rho}{N}\cdot\frac{1}{c-B-\delta}\left(\frac{N}{\rho} - \frac{A}{(B-\gamma)\delta}\right),$$

$$C_3 = (1-\epsilon_s)\frac{c\rho}{N}\cdot\frac{A}{(B-\gamma)\delta(c-\delta-\gamma)}.$$

The duration of the first phase of viral decline, denoted by D_1, is the time at which two curves $\log_{10}(C_1 e^{-ct})$ and $\log_{10}[C_2 e^{-(B+\delta)t}]$ intersect. Thus, we have $D_1 = \frac{\ln(\frac{C_1}{C_2})}{c-(B+\delta)}$.

Similarly, we have for the duration of the second phase of viral decline $D_2 = \frac{\ln(\frac{C_2}{C_3})}{B-\gamma}$.

If ϵ_s is close to 1, then $C_1 >> C_2$ and there is a visible first-phase viral decline with slope c. If ϵ_α is close to 1, then $A = (1-\epsilon_\alpha)\alpha$ is very small. As a consequence, $C_2 >> C_3$ and there is a visible second-phase viral decline with slope $B+\delta$.

6. Effect of κ on viral decline.

The effect of κ on the viral load decline is shown in the following figure. In panel A, we assume $\epsilon_s = \epsilon_\alpha = 0.99$. The viral load decline has three phases. As κ increases, the slope of the second-phase viral decline increases, whereas its duration decreases. In panel B, we assume $\epsilon_s = 0$ and $\epsilon_\alpha = 0.99$. The viral load decline has two phases. The phase with slope c is not visible. As κ increases, the slope of the first-phase viral decline increases.

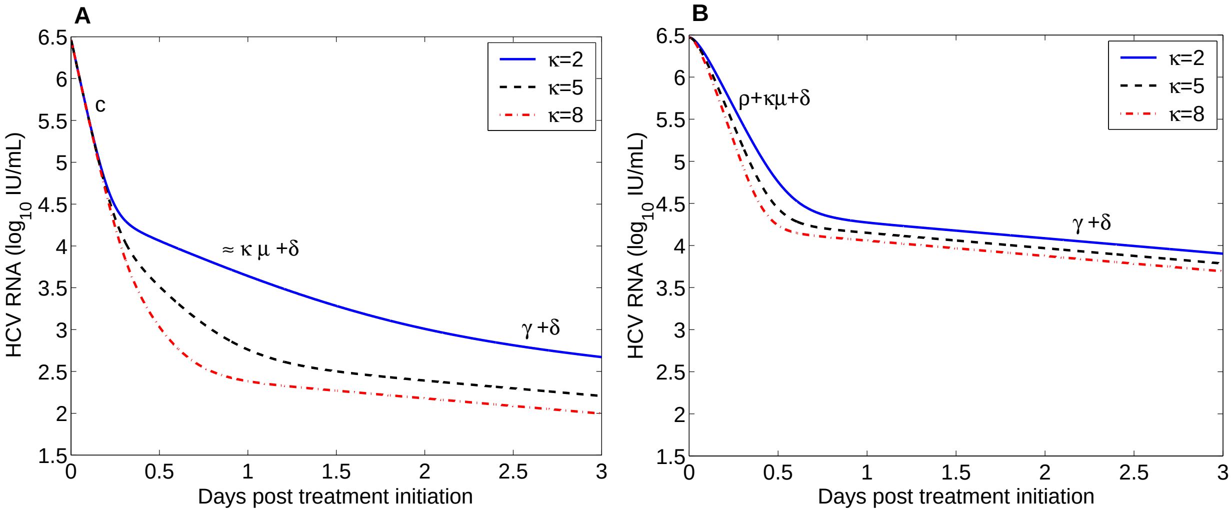

Figure S1. The effect of κ on viral decline. A. We assume $\epsilon_s = \epsilon_\alpha = 0.99$. **B**. We assume $\epsilon_s = 0$ and $\epsilon_\alpha = 0.99$. The other parameter values are the same as those in Figure 3 in the main text.

References

1. Rong L, Feng Z, Perelson, AS (2007) Mathematical analysis of age-structured HIV-1 dynamics with combination antiretroviral therapy. SIAM J Appl Math 67:731-56.

2. Lin K, Kwong AD, Lin C (2004) Combination of a hepatitis C virus NS3-NS4A protease inhibitor and alpha interferon synergistically inhibits viral RNA replication and facilitates viral RNA clearance in replicon cells. Antimicrob Agents Chemother 48:4784-92.

3. Guedj J, Neumann AU (2010) Understanding hepatitis C viral dynamics with direct-acting antiviral agents due to the interplay between intracellular replication and cellular infection dynamics. J Theor Biol 267:330-40.
